# Gas-Forming Liver Abscess versus Emphysematous Hepatitis: A Radiologic Diagnostic Dilemma—A Case Report and Review of the Literature

**DOI:** 10.1155/2019/5274525

**Published:** 2019-07-16

**Authors:** Youssef Ghosn, Ali Abdallah, Mohammed Hussein Kamareddine, Amine Geahchan, Ahmad Baghdadi, Ziad El-Rassi, Abbas Chamseddine, Raja Ashou

**Affiliations:** ^1^Faculty of Medicine and Medical Sciences, University of Balamand, Achrafieh, Beirut, Lebanon; ^2^Department of Diagnostic Radiology, Saint George Hospital University Medical Center, University of Balamand, Achrafieh, Beirut, Lebanon; ^3^Department of General Surgery, Saint George Hospital University Medical Center, University of Balamand, Achrafieh, Beirut, Lebanon

## Abstract

A 38-year-old diabetic woman, with history of cholecystectomy and ventral hernia repair, was hospitalized due to sudden-onset abdominal pain and fever. Computed tomography revealed a mixed collection containing necrotic debris and emphysematous change in the left lobe of the liver mainly in segments II and III. These radiological findings suggested emphysematous hepatitis (EH). The patient's condition deteriorated rapidly, and she was rushed to the operating room for urgent exploratory laparotomy where debridement was performed. Intraoperatively the patient was found to have an abscess with incomplete capsule concurrent with hepatic necrosis suggesting the co-occurrence of abscess and EH. The patient survived and was discharged after 13 days. Relevant literature was reviewed, and to the best of our knowledge, EH is an extremely rare entity with limited data regarding its pathogenesis, causative organisms, and management. EH is a rapidly invasive disease process that can be fatal if appropriate therapeutic intervention is delayed. Initial presentations are usually subtle, thus high clinical and radiological suspicion is required for early diagnosis and management to decrease associated mortality and morbidity. We hence report the first successfully treated case of EH with review of the literature.

## 1. Introduction

Intra-abdominal emphysematous parenchymal infections have been widely recognized involving a variety of abdominal organs, including the urinary tract, gallbladder, uterus, stomach, and pancreas. However, few reported cases have described similar emphysematous changes occurring in the liver [1, 2]. Although the spectrum of causes of hepatic gas detected with computed tomography (CT) is broad, emphysematous parenchymal involvement of the liver is extremely rare [3]. The first case of replacement of the liver parenchyma by air with no evidence of abscess was reported by Blachar et al. (2001) [4]. The diagnosis of this condition requires high clinical suspicion for a hepatobiliary pathology coupled with imaging and the ability of radiologists to identify the pathological process. Emphysematous hepatitis (EH) shares some radiological and clinical features with other entities caused by acute necrotizing, gas-forming infection such as emphysematous pyelonephritis [5]. However, EH is still poorly characterized and could be hard to differentiate from gas-forming pyogenic liver abscess (PLA). We herein report a case of EH in a diabetic, 38-year-old female, successfully treated with antibiotics and rapid surgical debridement. Unfortunately, all other reported cases with EH have led to fatality within one to three days of admission. A complete review of the literature is also presented.

## 2. Case Presentation

A 38-year-old woman was admitted to the Medical Intensive Care Unit (MICU) at Saint Georges University Hospital, Beirut, because of a 2 day history of chills and fever (reaching 39.5 degrees Celsius), along with nausea and vomiting (2 episodes), and 1 day history of severe abdominal pain that was diffuse and did not respond to symptomatic treatment. Patient then noted abdominal fullness, so she presented to our emergency department. The patient had a past medical history remarkable for gestational diabetes mellitus and a past surgical history of two C-sections, cholecystectomy (4 years prior to presentation), and most recently surgical repair of a ventral hernia with a mesh placement (4 years prior to presentation).

On presentation, the patient was awake, alert, and oriented to time, place, and person. However, the patient was in severe pain and acutely ill in appearance. She was febrile 38.9 C, but the rest of her vitals were stable. Examination was remarkable for mild scleral icterus, right upper quadrant tenderness, abdominal rigidity, and guarding, but the liver and spleen were not palpable. Initial complete blood count showed leukocytosis of 54.9 x109/L with 88.3% neutrophils, anemia with hematocrit of 26.1 % and hemoglobin of 8.3 g/dL, and platelet count of 280 x103/mL. C-reactive protein (CRP) was remarkably elevated measuring 49.33 mg/L.

Blood urea nitrogen was 53 mg/dL and creatinine 0.66 mg/dL. Liver function tests showed aspartate aminotransferase (AST) of 434 u/L, alanine aminotransferase (ALT) of 296 u/L, alkaline phosphatase of 133 u/L, gamma-glutamyltransferase of 29 u/L, total bilirubin of 4.28 mg/dL, and direct bilirubin of 1.89 mg/dL. Coagulation studies showed a prolonged prothrombin time (PT) of 15.44 seconds, with International Normalized Ratio (INR) of 1.29, and partial thromboplastin time (PTT) of 39.49 seconds. The rest of the serum biochemistry panel showed normal pancreatic enzymes, increased serum glucose of 281 mM, decreased total protein of 6 g/dL, and albumin of 3.4 g/dL.

**Figure 1:**
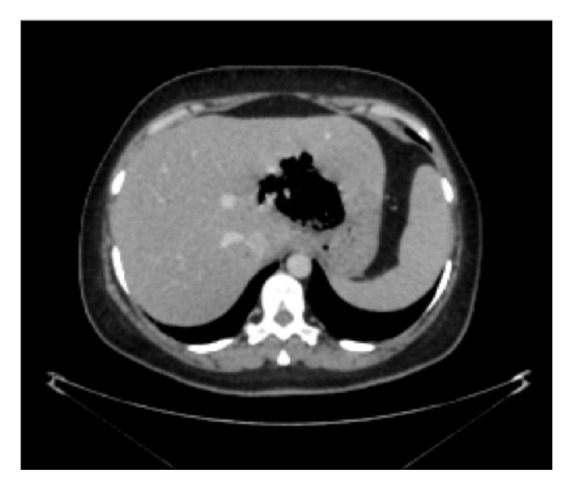
Axial abdominal CT scan with oral and IV contrast. Mixed collection containing air and debris spreading at the posterior aspect of the left lobe of the liver mainly in segments II and III, limited laterally by the left portal vein and extending medially towered the lesser curvature of the stomach associated with moderate intra- and extrahepatic biliary dilation. Sharply marginated left borders are noted.

**Figure 2:**
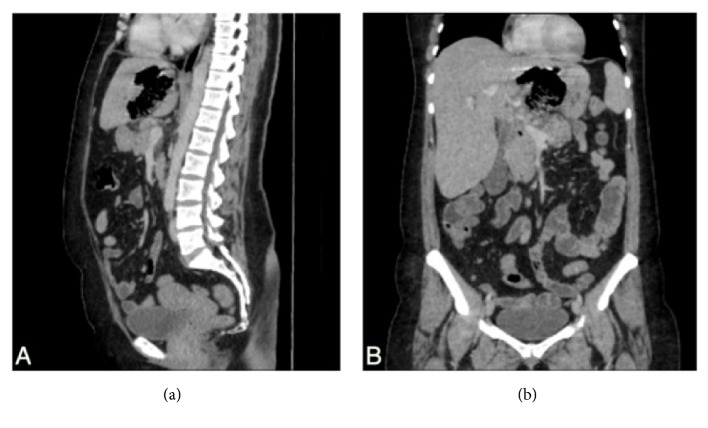
Sagittal (a) and coronal (b) abdominal CT scan with oral and IV contrast. Mixed collection containing air and debris measuring 8x7x5.5 cm is present. The lesion is spreading at the posterior aspect of the left lobe at segments II and III. Emphysematous changes of the adjacent parenchyma are noted.

As for the imaging done, chest X-ray was unremarkable. However, abdominal CT scan with oral and IV contrast demonstrated a mixed collection containing air and debris measuring 8x7x5.5 cm limited laterally by the left portal vein and extending medially toward the lesser curvature of the stomach associated with moderate intra- and extrahepatic biliary dilation. Moreover, it is spreading at the posterior aspect of the left lobe of the liver mainly in segments II and III. Emphysematous alveolarization of the adjacent hepatic parenchyma was also noted (Figures 1 and 2). The absence of cluster signs, septal breakage, collections in favor of pus, fluid collections and the lack of capsular enhancement of an abscess capsule made EH more likely than PLA.

The patient underwent urgent exploratory laparotomy. Preoperative Amoxicillin-Clavulanic acid was given. A huge cavity in the hepatic parenchyma was noted involving segments II and III along with necrotic liver parenchyma which underwent debridement. Exploration of the abdomen was done, with no other collection or pathology found. Liver necrotic tissue was sent to pathology and fluid was sent for culture. Pathology results showed liver parenchyma with marked mixed inflammatory prominent necrosis and focal accumulation of pus, with no evidence of granulomas or malignancy. Fluid culture grew* Escherichia coli* and* Enterococcus faecium*, so the patient was started on tigecycline. The surgery was uneventful and the patient was transferred to the ICU for monitoring and further management. Patient was stable in the ICU and her lab results improved significantly, so she was transferred to the floor and discharged home 13 days after her presentation.

## 3. Review of the Literature

### 3.1. Causes and Differential Diagnosis 

EH seems to share similar risk factor with other manifestations of gas-producing infections which are related to anaerobic bacteria and usually associated with immunosuppressive pathologies such as diabetes mellitus (DM) and cancer. Interestingly, most of these patients had history of recent abdominal surgery.

DM appears to be highly associated with EH. Similar to our case, the first case reported by Blachar et al. (2001) was of a patient with DM. This 43-year-old woman had short gut syndrome with the most recent bowel resection preformed 6 weeks before presentation [4]. Another case by Chauhan U et al. (2012) describes fulminant EH in a diabetic patient with replacement of the entire liver parenchyma by air [6].

Other than diabetes, gastrointestinal cancers seem to be associated with EH. One case reported by Kim JH et al. (2012) Showed EH in an 80-year-old patient with hilar cholangiocarcinoma [1]. Similarly, Letourneau-Guillon et al. (2010) reported a case of EH in 53-year-old patient with cholangiocarcinoma three months after undergoing left hepatectomy with hepaticojejunostomy and left hepatic duct extension [7]. Another case by Nada KM el al. (2017) reported EH in a patient with a history of pancreatic cancer and liver metastasis who underwent Whipple procedure 8 month prior to presentation [2].

Because EH is commonly related to liver infarction and infection, the pathophysiology of EH is thought to be due to superinfection of infarcted liver parenchyma [3]. It is usually caused by bacterial pathogens associated with emphysematous infections such as* Streptococcus mutans*,* Enterococcus faecalis* [2],* E. coli*,* Klebsiella*[8],* Enterobacter*,* Pseudomonas*, and* Proteus* [9] causing acid fermentation from tissue necrosis that ultimately forms a mixture of gases that could not be transported out of the liver due to the necrotic tissue [5, 10].

The differential diagnosis for gas in the liver is broad. Emphysematous changes in the liver has typically been seen in clinical situations involving gas-forming bacteria [11] and bowel infarction or following invasive procedures or therapeutic interventions such as sphincterotomy [12], hepatic artery thrombosis after liver transplantation (caused by damage of hepatic circulation) [13], radiofrequency ablation, and percutaneous ethanol injection in hepatocellular carcinoma [14]. Gas gangrene in the liver, which necessitates compromise of both the portal and arterial supply, has been reported in the setting of liver transplantation and hepatic trauma [13].

### 3.2. Clinical Manifestation and Outcome

Initial clinical manifestations are usually subtle and progress rapidly in the absence of therapeutic interventions [2]. Symptoms and signs include sudden-onset right upper abdominal pain, hepatomegaly, icterus, and altered mental status fever with eventual progression to septic shock [1, 2]. Lab values generally show leukocytosis and elevated LFTs [1, 2, 4, 6].

This entity has significant poor outcome, since most patients with this severe infection do not improve despite application of aggressive management (antibiotic therapy with or without drainage) and progress rapidly. The clinical outcome of EH appears to be fatal, as seen in all other reports, with death between one to three days after presentation [1, 2, 4, 6, 7]. Therefore, a more aggressive therapeutic modality is emphasized and surgical intervention should not be delayed in patients with EH in order to avoid total emphysematous replacement of hepatic parenchyma leading to death.

### 3.3. Radiological Features

When emphysematous liver changes are noted, it is important to check whether the gas is located in the bile ducts, liver parenchyma, vascular structures, or the lymphatic system in order to determine the origin. When the gas is located in the liver parenchyma, we should think of an infectious process like a liver abscess due to septic emboli, ascending cholangitis, or EH. Once gas is present in the liver parenchyma, it can spread through the hepatic veins, bile ducts, or the lymphatic system [3].

Although these two entities can present similarly, it important to differentiate between PLA and EH; however, EH is a new characterized entity with limited cases to be able to form definitive radiological diagnostic criteria. The difference between EH and gas-forming pyogenic liver abscesses is that PLA usually has clustered or multiseptated lesion (cluster signs and septal breakage) generally with pus and fluid collections [1, 8]. Often there is a double target sign due to the enhancement of the abscess capsule and the surrounding edema in the liver parenchyma [3]. In few cases of gas-forming PLA, air-fluid level or air bubbles can be present. On the other hand, in EH, the affected liver is totally replaced by air with no fluid content [1].

Moreover, in EH the arterial and portal venous blood supply is impaired causing sharply marginated wedge shaped areas, corresponding to the liver segments without contrast enhancement. In these infarcted liver segments, the parenchyma is replaced by gas which can further spread through the hepatic veins, biliary tract, or the lymphatic system [3].

For now, it seems that the definitive diagnosis requires surgical exploration. Our example demonstrates radiological features of EH; however, upon surgical exploration both entities (abscess and EH) were present, suggesting that both could be co-manifested with the probability that PLA contributed to EH.

## 4. Conclusion

EH is rare clinical entity with poor characterization. It is important to distinguish between EH and gas-forming PLA, knowing that both entities might be co-manifested. Patients should be treated with urgent exploratory laparotomy with surgical debridement. Appropriate antibiotics should be administered. For now, this is the only successfully treated case of EH.
